# Isovaleric Acidemia: A Rare Case of an Inborn Error of Metabolism

**DOI:** 10.7759/cureus.7150

**Published:** 2020-02-29

**Authors:** Adnan Khan, Bakhtyar Zahid, Sarbiland Khan, Samreen A Ahmad

**Affiliations:** 1 Pediatrics, Rehman Medical Institute, Peshawar, PAK; 2 Internal Medicine, Rehman Medical Institute, Peshawar, PAK

**Keywords:** isovaleric acidemia, metabolic acidosis

## Abstract

Isovaleric acidemia (IVA) is an autosomal recessive disease of the leucine metabolism due to a deficiency of isovaleryl-CoA dehydrogenase (IVD). We report the case of a six-month-old girl admitted with a seven-day history of fever, cough, stridor, vomiting, and respiratory distress. Second-degree consanguinity was documented between the parents. Urine organic acid analysis by gas chromatography-mass spectrometry showed marked excretion of 3-hydroxybutyric acid along with moderate excretion of 3- hydroxy-isovaleric acid. Isovaleric acidemia was diagnosed based on history, examination, and laboratory evaluation. The patient managed with fluid resuscitation, correction of her metabolic acidosis, antibiotics, and supportive care.

## Introduction

Isovaleric acidemia (IVA) is an autosomal recessive hereditary metabolic disorder caused by the inadequacy of isovaleryl-CoA dehydrogenase (IVD). IVD is an enzyme positioned proximally in the catabolic pathway of the essential amino acid leucine. Lack of IVD enzymes leads to an accumulation of isovaleric acid, 3-hydroxyisovaleric acid, isovaleryl (C5)-carnitine (IVC), and isovalerylglycine (IVG) [1].

IVA exists in two forms, acute and chronic intermittent. In both patterns, an acute episode of metabolic decompensation may appear during a catabolic state such as infection. Upper respiratory tract infections or excessive intake of high protein food can trigger the episodes. An acute type manifests as vomiting and severe acidosis during the beginning of life, with subsequent progression to lethargy, convulsion, coma, and, ultimately, death if not treated properly [2]. The characteristic odor of sweaty feet or rancid cheese odor may be present [1]. In the milder chronic intermittent pattern, the first clinical presentation may not be present until the child is a few months or a year old.

## Case presentation

A six-month-old girl was admitted to the pediatric unit with fever, cough, stridor, vomiting, and respiratory distress for seven days. She had previously been hospitalized multiple times and had required intensive care during the two previous hospitalizations. Of note, when the patient was not ill, she was healthy and noted to be asymptomatic. Birth history was uneventful, but one sibling died due to the same condition. Second-degree consanguinity was documented between the parents.

**Figure 1:**
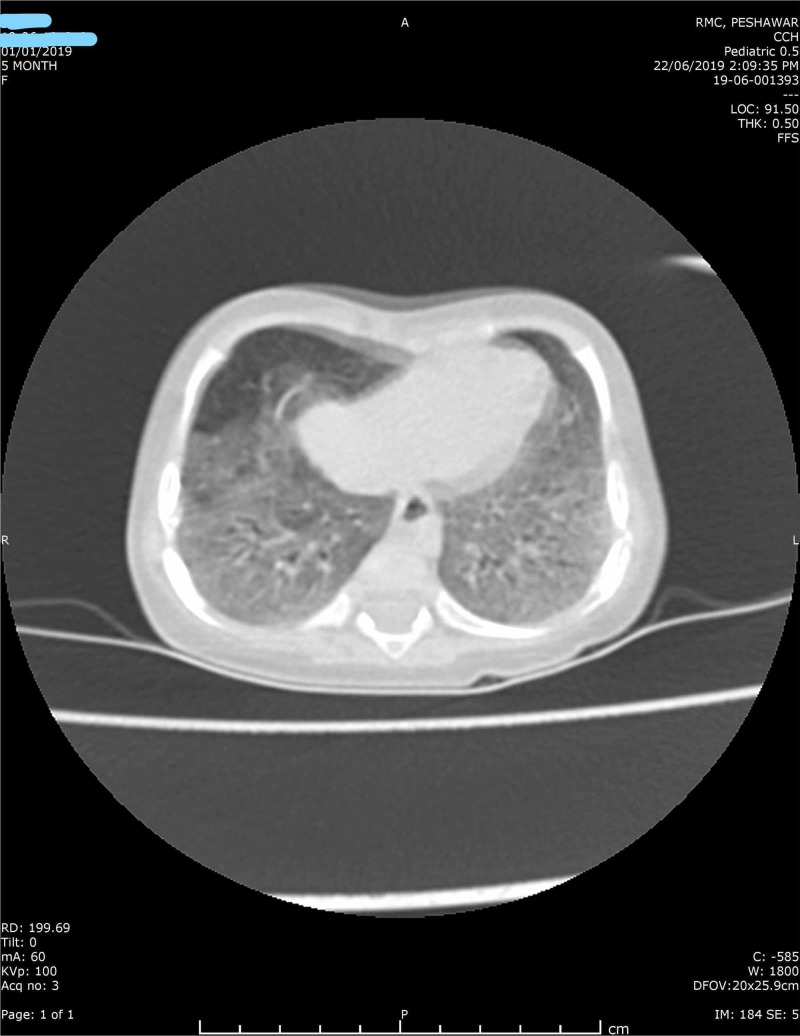
Computed tomography (CT) scan of the chest showing bilateral ground-glass appearance

On initial assessment, the patient was lethargic and dehydrated with marked Kussmaul breathing. Blood gas analysis revealed metabolic acidosis (pH 7.22, partial pressure of carbon dioxide (PCO2) 14.6 mmHg, bicarbonate (HCO_3_; 5.9 mmol/l) with a high anion gap (20.7 mmol/l) and an increased base excess (-19.1 mmol/l). Computed tomography (CT) chest showed bilateral ground-glass appearance (infective) (Figure 1), complete blood count (CBC) showed a high total leukocyte count (TLC) of 37, mostly neutrophil (60%), and her C-reactive protein (CRP) was 1.56 mg/dl. The results of the biochemical analysis were within normal limits (ammonia 37 umol/l). Ketonuria was also present (ketone 3+ / 300 mg/dl).

The patient was suspected to have bronchopneumonia with concern for underlying metabolic disorder. The patient was initiated on antibiotic ceftriaxone 50 mg/kg/day. Repeat arterial blood gas (ABG) revealed continued metabolic acidosis and the patient was initiated on a continuous bicarbonate infusion. Subsequent ABGs after the infusion of bicarbonate showed an improvement in her metabolic acidosis. Plasma amino acids and urine for organic acids were sent to evaluate for underlying metabolic disorders. The patient was also initiated on oral sodium bicarbonate, biotin, vitamin B12, and protein-free milk, and mother's milk was stopped after consultation with the chemical pathologist. The patient improved with the treatment and was shifted to the ward. She was initially on nasogastric feed and gradually oral feed was established. Repeated CBC showed hemoglobin of 6 mg/dl and red cell concentrates (RCC) was transfused at 10ml/kg during the treatment.

Urine organic acid analysis by gas chromatography-mass spectrometry showed marked excretion of 3-hydroxybutyric acid along with moderate excretion of 3-hydroxy-isovaleric acid, the diagnosis of isovaleric acidemia was made and the patient was sent home with medication and a scheduled follow-up.

## Discussion

Isovaleric acidemia (IVA) is an inborn error of leucine metabolism and is autosomal recessive, which can result in serious mortality and morbidity [3]. It was the earliest organic acidemia acknowledged in humans. A 2011 review of 176 cases found that diagnoses made early in life were associated with more drastic disease and a mortality of 33% while a lower mortality rate was noted in children diagnosed later with milder symptoms [4].

Patients may be developmentally normal but some mild to severe developmental delay has been reported [5]. A study by Grunert et al. described that the most common trigger is infection though an excess of protein or surgery did not play a significant function as a precipitating factor [4]. The first attack of a metabolic crisis in our patient occurred at the age of two months and was triggered by pneumonia.

Another study conducted by Sidbury et al. noted that four children of a second-cousin marriage died in the first week of lives with symptoms of convulsion, lethargy, dehydration, and an unusual urinary odor like sweaty feet which started after the third day of birth [6]. This case, born to a second-degree consanguineous marriage, presents with a recurrent illness like lethargy, dehydration, and one episode of convulsion and had an unusual odor of the body during acute illness. The unusual odor was due to butyric and hexanoic acids.

If a patient is not treated on time, the patient may proceed to coma and, ultimately, death because of cerebral edema or cerebral hemorrhage [7]. In our case, the patient was diagnosed very early, and appropriate treatment was started on time, so neurologically as well as physically, the patient is doing well to date. Regarding routine follow-up visits, there is no established laboratory marker for monitoring therapeutic control or disease state. Weight gain, growth, and development should be age-appropriate and, thus, body measurements are key parameters to follow on a routine basis. Specifically, protein malnutrition must be avoided if the patient is protein restricted.

## Conclusions

This present case report illustrates that organic acidemia should be kept in mind in the differential diagnosis when patients present with periodic vomiting, lethargy, respiratory distress, and are products of a consanguineous marriage, though the patient may be normal in between episodes.
